# Naloxone protection, social support, network characteristics, and overdose experiences among a cohort of people who use illicit opioids in New York City

**DOI:** 10.1186/s12954-022-00604-w

**Published:** 2022-03-04

**Authors:** Alex S. Bennett, Joy Scheidell, Jeanette M. Bowles, Maria Khan, Alexis Roth, Lee Hoff, Christina Marini, Luther Elliott

**Affiliations:** 1 School of Global Public Health, New York University, New York, USA; 2 Center for Drug Use and HIV/HCV Research, New York University, New York, USA; 3 Center for Opioid Epidemiology and Policy, Grossman School of Medicine, New York University, New York, USA; 4 Centre for Drug Policy and Evaluation, Unity Health Toronto, 209 Victoria St, Toronto, ON M5B 1T8 Canada; 5 Dornsife School of Public Health, Drexel University, Philadelphia, PA USA

**Keywords:** Naloxone protection, Social networks, Non-fatal overdose, Opioids

## Abstract

**Background:**

Despite increased availability of take-home naloxone, many people who use opioids do so in unprotected contexts, with no other person who might administer naloxone present, increasing the likelihood that an overdose will result in death. Thus, there is a social nature to being “protected” from overdose mortality, which highlights the importance of identifying background factors that promote access to protective social networks among people who use opioids.

**Methods:**

We used respondent-driven sampling to recruit adults residing in New York City who reported recent (past 3-day) nonmedical opioid use (*n* = 575). Participants completed a baseline assessment that included past 30-day measures of substance use, overdose experiences, and number of “protected” opioid use events, defined as involving naloxone and the presence of another person who could administer it, as well as measures of network characteristics and social support. We used modified Poisson regression with robust variance to estimate unadjusted and adjusted prevalence ratios (PRs) and 95% confidence intervals (CIs).

**Results:**

66% of participants had ever been trained to administer naloxone, 18% had used it in the past three months, and 32% had experienced a recent overdose (past 30 days). During recent opioid use events, 64% reported never having naloxone and a person to administer present. This was more common among those: aged ≥ 50 years (PR: 1.18 (CI 1.03, 1.34); who identified as non-Hispanic Black (PR: 1.27 (CI 1.05, 1.53); experienced higher levels of stigma consciousness (PR: 1.13 (CI 1.00, 1.28); and with small social networks (< 5 persons) (APR: 1.14 (CI 0.98, 1.31). Having a recent overdose experience was associated with severe opioid use disorder (PR: 2.45 (CI 1.49, 4.04), suicidality (PR: 1.72 (CI 1.19, 2.49), depression (PR: 1.54 (CI 1.20, 1.98) and positive urinalysis result for benzodiazepines (PR: 1.56 (CI 1.23, 1.96), but not with network size.

**Conclusions:**

Results show considerable gaps in naloxone protection among people who use opioids, with more vulnerable and historically disadvantaged subpopulations less likely to be protected. Larger social networks of people who use opioids may be an important resource to curtail overdose mortality, but more effort is needed to harness the protective aspects of social networks.

## Background

The United States (U.S.) remains in a public health emergency of opioid-related morbidity and mortality, with over 100,000 lives lost in 2021 [1, 2]. One of the main public health responses to this epidemic of overdose fatalities has been the widespread implementation of take-home naloxone (THN) programs [3–6]. In New York City (NYC), the setting for this study, naloxone is now widely available at pharmacies, drug treatment programs, syringe service programs (SSPs) [7, 8], mail order, shelters, prisons, jails, and through many community-based programs [9]. THN programs equip people who use drugs and interested community members with both naloxone and the knowledge to prevent (e.g. through avoiding concurrent use of opioids and alcohol or benzodiazepines) and respond to overdose events. Despite widespread access to THN for people who use drugs in places like NYC, there remain important open questions about the contexts in which THN availability does not equate to greater protection from overdose death.

THN overdose protection is contingent on consuming opioids in the presence of another person equipped with and capable of administering intranasal or intramuscular naloxone, as it cannot presently be reliably self-administered. As such, consuming drugs alone significantly heightens risk for overdose death [10]. Research examining the settings of overdose found that up to 75% of overdose fatalities in NYC occurred in private residences [11], placing the burden of responsibility for response on others in the household, if any exist. Ideally, others in the household would be trained in THN programs, observe or be aware of drug consumption events, and possess naloxone. Most people who witness overdoses are other people who use drugs [5]. However, even among people who use drugs and are trained in THN programs, they do not always carry naloxone. To illustrate, a study from Baltimore found that among longtime heroin users, two-thirds reported ever receiving naloxone, but of those only one-quarter reported always carrying it [12]. Existing research has identified many reasons why people who use drugs might not carry naloxone, thus limiting overdose protectedness, which include: stigma [13], ambivalence [13], insecurity about competence to use it [14], fear of police involvement, concerns about unnecessarily precipitating withdrawal [15], or consuming drugs alone. For people who primarily consume drugs alone, outside of networks of other people who use drugs and share the responsibility of overdose response, the value of THN is effectively negated.

There is a range of reasons why people might consume drugs alone, including the desire to avoid the stigmatizing judgment of others, safety, comfort, distrust of others, being pressured to share drugs, previous harmful experiences particularly when over-sedated, and drug use preferences [13, 16–18]. Using alone denies individuals the benefits identified with membership in social networks, which include: facilitating collective drug purchasing, group safety, resource sharing, maintaining group surreptitiousness, and social, economic and emotional support [17, 19]. Research has demonstrated how networks of people who use drugs can be resources for one another helping prevent many drug-related harms, thus creating a “social capital” that facilitates overdose response [20, 21]. Accordingly, the size of one’s social network of people who use drugs might be a critical indicator of their protectedness against an overdose fatality. To that end, the present study aims to uncover various factors associated with a person’s opioid-using network size and its relationship to their number of opioid use events during which another person and naloxone were present, thus representing a form of “protected use” from fatal overdose.

## Methods

### Study design and sample

To begin to explore the relationship between different types of social network supports and naloxone availability during overdose events, we developed a measure of overdose protectedness, reflecting the proportion of opioid use events in the past 30 days in which both a trusted person and naloxone were available. In the current analysis, we present descriptive statistics related to THN training and possession histories and examined potential associations between network characteristics, overdose protectedness, and non-fatal opioid-involved overdose among 575 people (*n* = 575) who use illicit opioids in NYC.

Participants enrolled in the study were persons aged 18 or older who were currently (defined as within the past 3 days) using an unprescribed opioid (e.g. heroin, fentanyl, and prescription opioids). Self-report of opioid use was verified using a rapid urinalysis tool from BTNX that included 9 fentanyl-class drugs in addition to heroin/morphine, benzodiazepines, alcohol, amphetamines, oxycodone, marijuana, and methadone metabolites. Recruitment followed a traditional respondent-driven sampling (RDS) approach [22, 23] that used numbered coupons to allow participants to refer up to three of their opioid-using network members to the study. Ten “seeds” representing ethnic, gender, and geographical diversity were established, ultimately resulting in a sample of 575 participants.

Once determined to be eligible and having provided informed consent, participants completed a face-to-face baseline survey instrument administered by a trained and experienced interviewer entering data on a tablet computer. Upon completion of baseline assessment, participants were trained in THN, which included a12-minute NYC Department of Health training video. Participants were compensated $60 for their time (approximately 2.5 h) and given 3 numbered coupons that they were asked to distribute to other people who use opioids. Participants were given an additional $15 for each referral of an eligible participant that resulted in a completed enrollment. Due to the COVID-19 pandemic, recruitment stopped in early March of 2020, roughly one week in advance of completing the target sample of *N* = 600. All procedures were approved by the Institutional Review Board of the host institution.

### Measures

#### Network size

Opioid-using network members were defined using the inclusion criteria for the study. We measured baseline network size using two indicators: 1) the participant-reported number of adults (> 18 years of age) whose names they knew (and who also knew their name) that live in NYC and were known to have used illicit opioids in the last three days, and 2) whom the participant had seen at least once in the past two weeks. Because the recruitment of participants was network-based, all participants had at least one network member (the person who had referred them to the study by providing an RDS coupon), and we considered those with less than five reported members in their opioid-using networks to have small networks. Among those who inject drugs (*n* = 102), we also measured the total number of people who had been in their presence when injecting drugs in the past three months, which we dichotomized as ≥ 1 versus 0 to capture isolated injection.

### Social support

Because the size of social networks is an imprecise indicator of how intimate or supportive these networks are, we included items about the frequency of reported forms of social and material support they had received from their non-drug-using (e.g., “your relatives, non-drug using friends and/or neighbors, program staff”) and drug-using network members in the past three months, including offers of a place to sleep, gifts of money with no strings attached, and emotional support when they were unhappy. We created separate dichotomous past 30-day indicators for each. To investigate how the constitution of networks may relate to overdose risk and protection, we also created a combined categorical support indicator defined as having received none of the forms of support from using and non-using networks; receiving ≥ 1 form of support from drug-using but no support from non-using network; receiving ≥ 1 form of support from non-using but no support from drug-using network; and receiving ≥ 1 form of support from both networks. Finally, participants reported if they were currently cohabiting with a romantic partner, cohabiting with anyone, and if that cohabiting person used opioids.

### Protection with naloxone

In the survey, participants reported the number of days they had used opioids in the past 30 days and the number of times they used, on average, each day, which we multiplied to estimate the total number of past 30-day opioid use events. Participants also reported the number of opioid use events in the past 30 days during which both naloxone and a person trusted to administer it were present, which are defined as “protected use” events. To calculate the proportion of protected use events in the past 30 days, we divided the reported number of opioid use events where naloxone and a person were present by the total reported number of opioid use events. We multiplied this proportion by 100 to capture the percentage of protected events, which we then dichotomized as never protected (e.g., 0% protected events) versus at least some protected events (e.g., > 0% protected events) in the past 30 days.

### Opioid overdose

Past-month indicators of opioid-related overdose were drawn from the Recent Overdose Experience Scale (RODES) [24]. Empirically-derived estimates suggest that less than 5% of heroin-related overdoses are fatal [25, 26] making non-fatal overdoses a critical outcome for research, particularly where sample size is constrained. The RODES assesses a number of clinical indicators of opioid-involved non-fatal overdose, including loss of consciousness, labored breathing, face or fingers turning blue, emergency services called, or naloxone administered, as well as asking respondents to estimate the number of days in the past month during which they believed they had an opioid-involved overdose, defined broadly as an event during which “you were more sedated drugged, or high than you wanted to be or felt was safe.” We considered an affirmative response to any of these experiences to indicate an overdose experience.

### Sample characteristics and covariates

Queried correlates included self-reported sociodemographic characteristics of age; race/ethnicity (categorized as non-Hispanic white, non-Hispanic Black, Hispanic/Latinx, and other e.g., Asian, American Indian or Native American, Native Hawaiian/Pacific Islander); sex (categorized as male/trans male and female/trans female); and employment status (categorized as employed full- or part-time, off books in informal/cash jobs, homemaker, or full-time student versus unemployed or unable to work for health reasons). Participants reported if they were currently homeless and if they had ever been incarcerated. Opioid use disorder (OUD) severity was measured using the Diagnostic and Statistical Manual of Mental Disorders Version 5 (DSM 5) criteria [27] and categorized as mild (score 2–3), moderate (score 4–5), or severe (score ≥ 6). Participants reported if they had ever injected heroin, as well as past 30-day use of cocaine, crack, marijuana, and/or benzodiazepine. Drug use-related stigma was measured using six items adapted from the Stigma Consciousness Scale [28], on which participants reported, using a Likert-type response scale, their level of agreement with statements such as, “I worry that other people might find out that I use drugs” and “I feel guilty about using drugs.” We summed responses to the six items (range 0–24, alpha = 0.76) and dichotomized at the sample median (scores ≥ 13). Posttraumatic stress disorder (PTSD) symptoms were measured using the six-item version of the PTSD Checklist [29] (range 0–24, alpha = 0.90), with scores ≥ 14 considered a positive screen for PTSD. Overall mental health was measured as a continuous score capturing the participants’ average across the 20 items in the DSM-5 Cross-Cutting Symptom scale (range 0–4; Cronbach’s alpha = 0.91); depression, anxiety, and suicidality were defined based on subscales [30]. The frequency with which participants visited a syringe exchange program (SEP) in the past three months was coded as “never,” “less than once a month,” and “almost daily/daily.” Participants reported if they were currently receiving treatment for opioid use and, if so, what form of treatment. Those receiving treatment were all receiving medication-assisted treatment, hence we created a dichotomous measure of no current treatment versus medication-assisted treatment. Finally, participants reported if they had ever been trained to administer naloxone and if they had administered it to anyone in the past three months.

### Analyses

All analyses were conducted in Stata 15.1 [31]. In univariate analyses, we estimated the frequency and proportion of sample characteristics in the total sample. In bivariate analyses, we estimated the frequency and proportion of having fewer than five members in one’s opioid network, of having no social support from using network members, of having 0% protected opioid use events, and of overdose experiences for each sample characteristic. We used a modified Poisson regression with robust variance estimation to estimate prevalence ratios (PRs) and 95% confidence intervals (CIs) for association. We used modified Poisson regression because our binary outcomes were not rare [32] and because this method provides statistically principled estimates when conducting regression analyses with data from an RDS study design [33]. For each indicator of network size, social support and cohabiting, we estimated the frequency and proportion of past 30 days: (1) events of using opioids without naloxone and a person present, and (2) having had an opioid overdose. We estimated unadjusted and adjusted PRs, with adjusted models controlling for age, sex, race/ethnicity, current homelessness, and OUD severity.

## Results

### Participant socio-demographic characteristics, drug use, and drug treatment and harm reduction access (Table 1)

**Table 1:** Participant characteristics in the total sample and by network characteristics (*N* = 575)

	*N* (%) in total sample^a^	*N* (%) with < 5 members in network^b^	PR (95% CI) for association with < 5 members in network^c^	*N* (%) with no support from using network members^d^	PR (95% CI) for association with no support from using network^e^
*Age*					
Less than 50 years of age	255 (44.3)	68 (26.8)	Ref	103 (40.6)	Ref
50 years of age or older	321 (55.7)	63 (19.6)	0.73 (0.54, 0.99)	156 (48.8)	1.20 (1.00, 1.45)
*Race/ethnicity*					
Non-Hispanic White	106 (18.4)	26 (24.8)	Ref	46 (43.4)	Ref
Non-Hispanic Black	217 (37.7)	47 (21.7)	0.87 (0.58, 1.33)	108 (49.8)	1.15 (0.89, 1.48)
Hispanic/Latinx	229 (39.8)	53 (23.1)	0.93 (0.62, 1.41)	97 (42.7)	0.98 (0.76, 1.28)
Other	19 (3.30)	4 (21.0)	0.85 (0.33, 2.16)	6 (31.6)	0.73 (0.36, 1.46)
*Sex*					
Female	193 (33.5)	43 (22.5)	Ref	90 (27.1)	Ref
Male	379 (65.8)	87 (23.0)	1.02 (0.74, 1.41)	167 (44.3)	0.94 (0.78, 1.13)
*Employment status*					
Unemployed	445 (77.3)	99 (22.3)	Ref	195 (44.0)	Ref
Employed	124 (21.5)	30 (24.2)	1.08 (0.76, 1.55)	62 (50.0)	1.14 (0.92, 1.39)
*Currently homeless*					
No	402 (69.9)	90 (22.4)	Ref	184 (45.9)	Ref
Yes	173 (30.1)	41 (23.7)	1.06 (0.76, 1.46)	75 (43.4)	0.76 (0.63, 0.93)
*Ever incarcerated*					
No	114 (19.8)	33 (29.0)	Ref	63 (55.8)	Ref
Yes	462 (80.2)	98 (21.3)	0.73 (0.52, 1.03)	196 (42.5)	0.59 (0.39, 0.89)
*Currently lives with romantic partner*					
No	426 (74.1)	103 (24.2)	Ref	192 (45.3)	Ref
Yes	149 (25.9)	28 (18.8)	0.78 (0.53, 1.13)	67 (44.7)	0.99 (0.80, 1.21)
*Currently lives with romantic partner who uses opioids*					
No	473 (82.3)	115 (24.3)	Ref	215 (45.6)	Ref
Yes	102 (17.7)	16 (15.7)	0.64 (0.40, 1.04)	44 (42.7)	0.94 (0.73, 1.20)
*Currently lives with anyone*					
No	140 (24.4)	30 (21.4)	Ref	67 (48.2)	Ref
Yes	434 (75.6)	101 (23.3	1.09 (0.76, 1.56)	192 (44.2)	0.92 (0.75, 1.12)
*Currently lives with anyone who uses opioids*					
No	399 (72.0)	98 (24.6)	Ref	186 (46.8)	Ref
Yes	155 (28.0)	27 (17.4)	0.71 (0.48, 1.04)	64 (41.0)	0.88 (0.70, 1.09)
*Current living situation*					
Lives alone	140 (25.3)	30 (21.4)	Ref	67 (48.2)	Ref
Not with anyone who uses opioids	259 (46.8)	68 (26.2)	1.22 (0.84, 1.79)	119 (46.1)	0.96 (0.77, 1.19)
Someone who uses opioids	155 (28.0)	27 (17.4)	0.81 (0.51, 1.30)	64 (41.0)	0.85 (0.66, 1.10)
*Length of opioid use history*					
15 years of less	154 (16.8)	46 (30.1)	Ref	71 (46.4)	Ref
16–26 years	136 (23.6)	30 (22.1)	0.73 (0.49, 1.09)	52 (38.2)	0.82 (0.63, 1.08)
27–35 years27–35 years	147 (25.6)	32 (21.8)	0.72 (0.49, 1.07)	72 (49.0)	1.06 (0.83, 1.34)
36 years or longer	137 (23.8)	23 (16.8)	0.56 (0.36, 0.87)	64 (47.1)	1.01 (0.79, 1.30)
*OUD severity*					
Mild/moderate	95 (16.5)	24 (25.3)	Ref	56 (59.0)	Ref
Severe	441 (76.6)	93 (21.1)	0.84 (0.57, 1.24)	186 (42.4)	0.72 (0.59, 0.88)
*Ever injected heroin*					
No	365 (63.4)	85 (23.3)	Ref	175 (48.2)	Ref
Yes	196 (34.0)	39 (20.0)	0.86 (0.61, 1.20)	75 (38.3)	0.80 (0.64, 0.98)
*Cocaine use in past 30 days*					
No	288 (50.0)	77 (26.7)	Ref	139 (48.4)	Ref
Yes	288 (50.0)	54 (18.8)	0.70 (0.52, 0.96)	120 (41.8)	0.86 (0.72, 1.04)
*Crack use in past 30 days*					
No	415 (72.0)	95 (23.0)	Ref	197 (47.6)	Ref
Yes	161 (28.0)	26 (22.4)	0.97 (0.70, 1.37)	62 (38.8)	0.81 (0.65, 1.01)
*Marijuana use in past 30 days*					
No	358 (62.2)	84 (23.5)	Ref	170 (47.6)	Ref
Yes	218 (37.8)	47 (21.6)	0.92 (0.67, 1.26)	89 (41.1)	0.86 (0.71, 1.04)
*Benzodiazepine use in past 30 days*					
No	387 (67.2)	88 (22.8)	Ref	178 (46.2)	Ref
Yes	189 (32.8)	43 (22.8)	1.00 (0.72, 1.38)	81 (42.9)	0.93 (0.76, 1.13)
*Fentanyl detected in urine analysis*					
No	268 (46.5)	64 (23.9)	Ref	135 (50.6)	Ref
Yes	306 (53.1)	66 (21.6)	0.90 (0.67, 1.23)	123 (40.3)	0.80 (0.66, 0.96)
*Oxycodone detected in urine analysis*					
No	541 (93.9)	123 (22.8)	Ref	247 (45.7)	Ref
Yes	33 (5.7)	7 (21.2)	0.93 (0.47, 1.83)	11 (34.4)	0.75 (0.46, 1.22)
*Heroin detected in urine analysis*					
No	79 (13.7)	17 (21.5)	Ref	43 (55.1)	Ref
Yes	495 (85.9)	113 (22.9)	1.06 (0.68, 1.67)	215 (43.5)	0.79 (0.63, 0.99)
*Stigma (median* ≥ *13)*					
No	278 (48.3)	53 (19.1)	Ref	120 (43.2)	Ref
Yes	280 (48.6)	68 (24.4)	1.27 (0.93, 1.76)	127 (45.5)	1.05 (0.88, 1.27)
*PTSD*					
No	513 (89.1)	117 (22.8)	Ref	232 (45.4)	Ref
Yes	62 (10.8)	14 (22.6)	0.99 (0.61, 1.61)	27 (43.6)	0.96 (0.71, 1.29)
Omnibus mental health*	0.7 (0.25, 1.3)		Ref		Ref
			1.02 (0.84, 1.24)		0.99 (0.87, 1.12)
*Depression*					
No	264 (45.9)	62 (23.5)	Ref	115 (43.6)	Ref
Yes	69 (54.1)	69 (22.2)	0.94 (0.70, 1.28)	114 (46.4)	1.07 (0.89, 1.28)
*Anxiety*					
No	314 (54.6)	70 (22.3)	Ref	143 (45.5)	Ref
Yes	216 (45.4)	61 (23.4)	1.05 (0.78, 1.42)	116 (44.6)	0.98 (0.82, 1.17)
*Suicidality*					
No	546 (95.1)	125 (22.9)	Ref	247 (45.2)	Ref
Yes	28 (4.9)	6 (21.4)	0.94 (0.45, 1.94)	12 (44.4)	0.98 (0.64, 1.51)
*SEP visits in past 3 months*					
No	347 (60.2)	88 (25.4)	Ref	172 (49.7)	Ref
Yes	170 (29.5)	30 (17.8)	0.70 (0.48, 1.02)	64 (17.9)	0.76 (0.51, 0.95)
*Currently opioid treatment*					
No current treatment	254 (44.2)	71 (28.0)	Ref	104 (41.1)	Ref
Medication assisted treatment	321 (55.8)	60 (18.7)	0.67 (0.49, 0.90)	155 (48.3)	1.17 (0.98, 1.41)
*Ever trained to administer naloxone*					
No	193 (33.5)	56 (29.0)	Ref	95 (49.5)	Ref
Yes	379 (65.8)	93 (19.3)	0.66 (0.49, 0.90)	163 (43.1)	0.87 (0.72, 1.05)
*Administered naloxone in past 3 months*					
No	474 (82.3)	120 (25.4)	Ref	227 (48.1)	Ref
Yes	102 (17.7)	11 (10.8)	0.42 (0.24, 0.76)	32 (31.4)	0.65 (0.48, 0.88)

^a^May not sum to 575 due to missing values

^b^*N* = 575; 5 members is the 25th percentile [131 (22.7%) with < 5 members; 444 (77.1%) with 5 + members]

^c^Compared to 5 + Members

^d^*N* = 575 [259 (45.0%) have no material or emotional support; 315 (54.7%) have at least one form of support]

^e^Compared to those with at least one form of support

^*^Median (IQR); scale is standardized

Of the study population of opioid users, 65.8% were men and 33.5% were women; their mean age was 48.3 years with 55.7% 50 years of age or older; and 39.8% were Hispanic/Latinx, 37.7% were non-Hispanic Black, 18.4% were non-Hispanic white, and 3.3% were categorized as another racial/ethnic background (Tables 1, 2). The majority of the sample were unemployed (77.3%)and had a history of incarceration (80.2%). Thirty percent of participants reported they were currently homeless. The majority of those not experiencing homelessness lived with someone else (75.6%), with one-quarter reporting cohabitation with a romantic partner (25.9%) and 17.7% reported cohabitating with a romantic partner used opioids. Over one-quarter cohabited some someone who uses opioids, including romantic partners (28.0%).

**Table 2:** Participant characteristics in the total sample and by unprotected use and OD experience in past 30 days (*N* = 575)

	*N* (%) with unprotected use	PR (95% CI) for association unprotected use	*N* (%) with OD experience	PR (95% CI) for association with OD experience^a^
*Age*				
Less than 50 years of age	146 (58.4)	Ref	105 (41.3)	Ref
50 Years of age or older	219 (68.6)	1.18 (1.03, 1.34)	80 (24.9)	0.60 (0.47, 0.77)
*Race/ethnicity*				
Non-Hispanic White	59 (56.2)	Ref	45 (42.9)	Ref
Non-Hispanic Black	153 (71.2)	1.27 (1.05, 1.53)	58 (26.7)	0.62 (0.46, 0.85)
Hispanic/Latinx	139 (61.5)	1.09 (0.90, 1.33)	78 (34.1)	0.79 (0.60, 1.06)
Other	11 (61.1)	1.09 (0.72, 1.63)	3 (15.8)	0.37 (0.13, 1.07)
*Sex*				
Female	117 (61.6)	Ref	58 (30.2)	Ref
Male	248 (66.1)	1.07 (0.94, 1.23)	123 (32.4)	1.07 (0.83, 1.39)
*Employment status*				
Unemployed	289 (65.5)	Ref	132 (29.7)	Ref
Employed	70 (57.8)	0.88 (0.75, 1.04)	53 (42.7)	1.44 (1.12, 1.84)
*Currently homeless*				
No	264 (66.7)	Ref	133 (33.2)	Ref
Yes	101 (58.4)	0.88 (0.76, 1.01)	52 (29.9)	0.90 (0.69, 1.18)
*Ever incarcerated*				
No	74 (66.7)	Ref	36 (31.6)	Ref
Yes	291 (63.5)	0.95 (0.82, 1.11)	149 (32.3)	1.02 (0.76, 1.38)
*Length of opioid use history*				
15 Years of less	89 (58.6)	Ref	66 (42.9)	Ref
16–26 years	88 (65.2)	1.11 (0.93, 1.34)	41 (30.2)	0.70 (0.51, 0.96)
27–35 years	92 (62.8)	1.07 (0.89, 1.29)	48 (32.6)	0.76 (0.57, 1.02)
36 years or longer	97 (71.3)	1.22 (1.03, 1.44)	30 (21.9)	0.51 (0.35, 0.74)
*OUD severity*				
Mild/moderate	67 (72.0)	Ref	14 (14.7)	Ref
Severe	271 (62.2)	0.86 (0.74, 1.00)	159 (36.1)	2.45 (1.49, 4.04)
*Ever injected heroin*				
No	255 (70.8)	Ref	107 (29.3)	Ref
Yes	98 (50.3)	0.71 (0.61, 0.83)	75 (38.5)	1.31 (1.03, 1.66)
*Cocaine use in past 30 days*				
No	196 (68.8)	Ref	82 (28.6)	Ref
Yes	169 (59.5)	0.86 (0.76, 0.98)	103 (35.8)	1.25 (0.98, 1.59)
*Crack use in past 30 days*				
No	265 (65.0)	Ref	121 (29.2)	Ref
Yes	100 (62.1)	0.96 (0.83, 1.10)	64 (39.8)	0.96 (0.83, 1.10)
*Marijuana use in past 30 days*				
No	235 (66.2)	Ref	96 (26.9)	Ref
Yes	130 (60.8)	0.92 (0.80, 1.05)	89 (40.8)	1.52 (1.20, 1.92)
*Benzodiazepine use in past 30 days*				
No	254 (66.5)	Ref	105 (27.2)	Ref
Yes	111 (59.4)	0.89 (0.78, 1.02)	80 (42.3)	1.56 (1.23, 1.96)
*Fentanyl detected in urine analysis*				
No	184 (68.9)	Ref	89 (33.2)	Ref
Yes	179 (59.7)	0.86 (0.76, 0.98)	95 (31.2)	0.94 (0.74, 1.19)
*Oxycodone detected in urine analysis*				
No	344 (63.9)	Ref	172 (31.8)	Ref
Yes	19 (65.5)	1.02 (0.78, 1.34)	12 (37.5)	1.18 (0.74, 1.88)
*Heroin detected in urine analysis*				
No	49 (62.0)	Ref	32 (40.5)	Ref
Yes	314 (64.3)	1.04 (0.86, 1.25)	152 (30.8)	0.76 (0.56, 1.02)
*Stigma (median* ≥ *13)*				
No	167 (60.5)	Ref	84 (30.3)	Ref
Yes	189 (68.5)	1.13 (1.00, 1.28)	96 (34.3)	1.13 (0.89, 1.44)
*PTSD*				
No	330 (65.0)	Ref	161 (31.4)	Ref
Yes	35 (57.4)		24 (38.7)	1.23 (0.88, 1.73)
Omnibus mental health*		Ref		Ref
		0.95 (0.86, 1.04)		1.30 (1.14, 1.48)
*Depression*				
No	172 (65.9)	Ref	66 (24.9)	Ref
Yes	193 (62.7)	0.95 (0.84, 1.08)	119 (38.4)	1.54 (1.20, 1.98)
*Anxiety*				
No	210 (67.5)	Ref	89 (28.2)	Ref
Yes	155 (60.1)	0.89 (0.78, 1.01)	96 (36.9)	1.31 (1.03, 1.66)
*Suicidality*				
No	349 (64.4)	Ref	170 (31.1)	Ref
Yes	16 (59.3)	0.92 (0.67, 1.27)	15 (53.6)	1.72 (1.19, 2.49)
*SEP visits in past 3 months*				
No	251 (73.4)	Ref	95 (27.4)	Ref
Yes	80 (47.3)	0.64 (0.54, 0.76)	64 (37.6)	1.38 (1.06, 1.78)
*Currently opioid treatment*				
No current treatment	161 (64.1)	Ref	99 (39.1)	Ref
Medication assisted treatment	204 (64.2)	1.00 (0.88, 1.13)	86 (26.7)	0.68 (0.54, 0.86)
*Ever trained to administer naloxone*				
No	162 (85.3)	Ref	51 (26.4)	Ref
Yes	200 (53.3)	0.62 (0.56, 0.70)	132 (34.9)	1.32 (1.00, 1.74)
*Administered naloxone in past 3 months*				
No	325 (69.3)	Ref	135 (28.5)	Ref
Yes	40 (40.0)	0.58 (0.45, 0.74)	50 (49.5)	1.74 (1.36, 2.22)

^a^May not sum to 575 due to missing values

^*^Median (IQR); scale is standardized

Based on DSM-5 criteria, the majority of the sample had severe OUD (76.6%), with 34.0% reporting lifetime history of heroin injection. Urine-based toxicology indicated 85.9% had recent exposure to heroin, 53.1% to fentanyl, and 5.7% to oxycodone. Substantial proportions reported use of other drugs in the past 30 days including cocaine (50.0%), crack (28.0%), marijuana (37.8%), and a benzodiazepine (32.8%). Participants reported experiencing drug-related stigma (48.6%), depressive symptoms (54.1%), anxiety symptoms (45.4%), PTSD (10.8%), and suicidal ideation (4.9%). Only 29.0% reported SEP use. Of the 575 recruited participants, 65.8% had ever been trained to use naloxone, and 17.7% had administered it in the past three months.

Of those trained to use naloxone (*n* = 379), 80% (302/379) were in possession of a THN kit at the time of the interview. On average, participants reported using 4.4 times (standard deviation [SD] 7.6) per day in the past 30 days and using on 23.6 days (SD 9.0) of the past 30 days; the mean number of use events in the days 30 days was 104.0 (SD 163.8). The mean number of days naloxone was reported to be available when using opioids in the past 30 days was 7.9 days. When asked how often naloxone was available during opioid use events in the past 30 days, 45% of all participants reported naloxone was never available during opioid use, 35% it was sometimes, and 20% reported it was always available.

### Network size and support by respondent characteristics (Table 1)

The prevalence of respondents with a small network (in the bottom 25^th^ percentile for number in the network; < 5 members) was 22.7% (Table 1). There was evidence that the prevalence of having a small network was lower in those who were 50 years or older (PR: 0.73; 95% CI 0.54, 0.99), who lived with either a romantic partner or any person who uses opioids (partner: PR: 0.64, 95% CI 0.40, 1.04; any person: 0.71, 95% CI 0.48, 1.04). Small network size also was less likely for those in MAT (PR: 0.67, 95% CI 0.49, 0.90) and among those who were trained in and had administered naloxone (trained: PR: 0.66, 95% CI 0.49, 0.90); (administered: PR: 0.42, 95% CI 0.24, 0.76). Network size was not correlated with gender, race/ethnicity, socio-economic factors, drug use patterns, or mental health factors.

The prevalence of respondents with no material or emotional support (from either drug using network members or non-drug using network members) was 45.0% (Table 1). A lack of support was more common among those aged 50 years and older (PR: 1.20; 95% CI 1.00, 1.45) (Table 1). Race/ethnicity, gender, and employment were not correlated with having or not having support. Lack of support was less common among respondents who were homeless (PR: 0.76; 95% CI 0.63, 0.93), those with a history of incarceration (PR = 0.59; 95% CI 0.39, 1.45), and those with severe OUD (PR: 0.72, 0.59, 0.88). Lack of support also was less likely among those who attended SEPs (PR: 0.76, 95% CI 0.51, 0.95) and who were trained in and had administered naloxone (trained: PR: 0.87, 95% CI 0.72, 1.05); (administered: PR: 0.65, 95% CI 0.48, 0.88).

### Overdose risk by respondent characteristics (Table 2)

When asked how often both naloxone and a person trusted to administer it were present during opioid use events in the past 30 days, 65% reported both naloxone and a person to administer it was never available during opioid use and hence were considered 0% protected from fatal overdose during those events. Having 0% overdose protection was more common among those aged 50 years and older (PR: 1.18, 95% CI 1.03, 1.34), non-Hispanic Black respondents (PR: 1.27, CI 1.05, 1.53) was less likely among opioid users who had severe OUD (PR: 0.86, 95% CI 0.74, 1.00), who injected heroin (PR: 0.71, 95% CI 0.61, 0.83), and who tested positive for fentanyl (95% CI 0.86, 95% CI 0.76, 0.98). There was also evidence that 0% protection was less common among those who used other substances including cocaine (PR: 0.86, 95% CI 0.76, 0.98) and benzodiazepines (PR: 0.89, 95% CI 0.78, 1.02) and who had anxiety symptoms (PR: 0.89, 95% CI 0.78, 1.01). Those who were trained in and who had administered naloxone also were less likely to be 0% protected during opioid use (trained: PR: 0.62, 95% CI 0.56, 0.70); (administered: PR: 0.58, 95% CI 0.45, 0.74).

A total of 185 (32%) had an OD experience in the past 30 days at baseline. Having experienced an overdose was less common among those aged 50 years and older (PR: 0.60, 95% CI 0.47, 0.77) and non-Hispanic Black respondents (PR: 0.62, 95% CI 0.46, 0.85) and was more common among those who were employed (PR: 1.44, 95% CI 1.12, 1.84). Overdose experience was more common among opioid users who had severe OUD (PR: 2.45; 95% CI 1.49, 4.04), and those who injected heroin (PR: 1.31; 95% CI 1.03, 1.66). There is also evidence that overdose experience was more common among those who used other substances including cocaine (PR: 1.25, 95% CI 0.98, 1.59), marijuana (PR: 1.52; 95% CI 1.20, 1.92) and benzodiazepines (PR: 1.56, 95% CI 1.23, 1.96). Overdose experience was strongly associated with suicidality (PR: 1.72, 95% CI 1.19, 2.49) and also was associated with symptoms of depression (PR: 1.54: 95% CI 1.20, 1.98) and anxiety (PR: 1.31, 95% CI 1.03, 1.66). Those who were trained in and who had administered naloxone also were more likely to have more overdose experiences (trained: PR: 1.32, 95% CI 1.00, 1.74); (administered: PR: 1.74, 95% CI 1.36, 2.22).Those who tested positive for fentanyl did not have elevated overdose experiences compared with those who tested negative (PR: 0.94; 95% CI 0.74, 1.19).

### Associations: network and support factors and 0% protection (lacks naloxone and/or person to administer it) during opioid use (Table 3)

**Table 3:** Associations between network and support factors and never protected with naloxone and a person to administer during opioid use events in past 30 days

	*N* (%) with no protection	PR (95% CI)	APR (95% CI)^a^
*Network size (injecting)*			
More than 1	64 (43.8)	Ref	Ref
0 in Network	38 (69.1)	1.58 (1.22, 2.03)	1.61 (1.23, 2.11)
*Network size (opioid-using)*			
More than 5	275 (62.5)	Ref	Ref
< 5 in network	90 (69.8)	1.37 (0.90, 2.09)	1.14 (0.98, 1.31)
*Network size (opioid using, excluding injectors)*			
More than 5	201 (71.3)	Ref	Ref
< 5 in Network	62 (72.10	1.01 (0.87, 1.18)	1.03 (0.88, 1.21)
*Non-using network gave place to stay in past 3 months*			
No	248 (65.1)	Ref	Ref
Yes	117 (63.2)	0.97 (0.85, 1.11)	1.08 (0.93, 1.24)
*Non-using network gave money in past 3 months*			
No	133 (62.7)	Ref	Ref
Yes	229 (64.9)	1.03 (0.91, 1.18)	1.07 (0.93, 1.22)
*Non-using network gave emotional support in past 3 months*			
No	80 (61.1)	Ref	Ref
Yes	281 (65.2)	1.06 (0.92, 1.24)	1.17 (0.99, 1.38)
*Using network gave place to stay in past 3 months*			
No	267 (65.4)	Ref	Ref
Yes	98 (61.2)	0.94 (0.81, 1.08)	1.03 (0.88, 1.20)
*Using network gave money in past 3 months*			
No	211 (68.5)	Ref	Ref
Yes	154 (59.5)	0.87 (0.76, 0.98)	0.85 (0.75, 0.98)
*Using network gave emotional support in past 3 months*			
No	144 (72.0)	Ref	Ref
Yes	221 (60.2)	0.84 (0.74, 0.94)	0.87 (0.76, 0.99)
*Support from networks*			
No support	41 (69.5)	Ref	Ref
At least one form of support from using	16 (48.5)	0.70 (0.47, 1.03)	0.70 (0.47, 1.04)
At least one form of support from non-using	174 (62.6)	0.98 (0.80, 1.19)	1.00 (0.82, 1.24)
At least one from of support from both	132 (68.0)	0.90 (0.74, 1.09)	0.95 (0.77, 1.16)
*Currently lives with romantic partner*			
No	277 (65.8)	Ref	Ref
Yes	88 (59.5)	0.90 (0.78, 1.05)	0.89 (0.76, 1.04)
*Currently lives with romantic partner who uses opioids*			
No	312 (67.0)	Ref	Ref
Yes	53 (51.5)	0.77 (0.63, 0.94)	0.77 (0.62, 0.95)
*Currently lives with anyone*			
No	92 (66.7)	Ref	Ref
Yes	272 (63.3)	0.95 (0.83, 1.09)	0.99 (0.85, 1.15)
*Currently lives with anyone who uses opioids*			
No	267 (68.1)	Ref	Ref
Yes	82 (52.6)	0.77 (0.66, 0.91)	0.79 (0.66, 0.94)
*Current living situation*			
Lives alone	92 (66.7)	Ref	Ref
Not with anyone who uses opioids	175 (68.9)	1.03 (0.89, 1.19)	1.06 (0.91, 1.24)
Someone who uses opioids	82 (52.6)	0.79 (0.65, 0.95)	0.82 (0.67, 1.01)

^a^Adjusted for age, sex, race/ethnicity, current homelessness, and OUD severity

#### Network size

In both unadjusted analyses and in multivariable analyses adjusting for age, sex, race/ethnicity, current homelessness, and OUD severity, among injectors, having no person in one’s injection network was associated with moderate increases in the prevalence of having no protection during opioid use events (APR: 1.61, 95% CI 1.23, 2.11) (Table 4). In the sample overall, when including injecting and non-injecting participants, the APR for the association between small network size (< 5 persons) and never being protected during opioid use was 1.14 (95% CI 0.98, 1.31).

**Table 4:** Associations between network and support factors and any OD experiences in past 30 days

	*N* (%) with OD experience	PR (95% CI)	APR (95% CI)^a^
*Network size (injecting)*			
More than 1	65 (44.5)	Ref	Ref
0 in Network	15 (27.3)	0.61 (0.38, 0.99)	0.80 (0.51, 1.26)
*Network size (opioid using)*			
More than 5	143 (32.3)	Ref	Ref
< 5 in Network	42 (32.1)	0.99 (0.75, 1.32)	1.04 (0.79, 1.36)
*Network size (opioid using, excluding injectors)*			
More than 5	79 (27.7)	Ref	Ref
< 5 in Network	26 (29.2)	1.05 (0.72, 1.53)	1.12 (0.78, 1.60)
*Non-using network gave place to stay in past 3 months*			
No	104 (26.9)	Ref	Ref
Yes	80 (43.0)	1.60 (1.26, 2.02)	1.42 (1.10, 1.83)
*Non-using network gave money in past 3 months*			
No	60 (27.9)	Ref	Ref
Yes	122 (34.3)	1.23 (0.95, 1.59)	1.05 (0.81, 1.36)
*Non-using network gave emotional support in past 3 months*			
No	29 (21.8)	Ref	Ref
Yes	152 (34.9)	1.60 (1.13, 2.27)	1.41 (0.99, 2.00)
*Using network gave place to stay in past 3 months*			
No	111 (26.9)	Ref	Ref
Yes	73 (45.3)	1.69 (1.34, 2.13)	1.52 (1.18, 1.95)
*Using network gave money in past 3 months*			
No	92 (29.4)	Ref	Ref
Yes	92 (35.4)	1.20 (0.95, 1.53)	1.22 (0.96, 1.55)
*Using network gave emotional support in past 3 months*			
No	54 (26.7)	Ref	Ref
Yes	129 (34.8)	1.30 (1.00, 1.70)	1.27 (0.96, 1.67)
*Support from networks*			
No support	8 (13.3)	Ref	Ref
At least one form of support from using	13 (39.4)	2.95 (1.36, 6.40)	2.79 (1.31, 5.93)
At least one form of support from non-using	57 (28.9)	2.17 (1.10, 4.29)	1.89 (0.97, 3.67)
At least one form of support from both	104 (37.1)	2.78 (1.43, 5.41)	2.30 (1.20, 4.39)
*Currently lives with romantic partner*			
No	133 (31.3)	Ref	Ref
Yes	52 (34.7)	1.11 (0.85, 1.45)	1.11 (0.95, 1.46)
*Currently lives with romantic partner who uses opioids*			
No	148 (31.4)	Ref	Ref
Yes	37 (35.9)	1.14 (0.86, 1.53)	1.00 (0.72, 1.39)
*Currently lives with anyone*			
No	41 (29.3)	Ref	Ref
Yes	144 (33.2)	1.13 (0.85, 1.51)	1.02 (0.75, 1.39)
*Currently lives with anyone who uses opioids*			
No	121 (30.4)	Ref	Ref
Yes	58 (37.2)	1.22 (0.95, 1.57)	1.09 (0.83, 1.44)
*Current living situation*			
Lives alone Lives alone	41 (29.3)	Ref	Ref
Not with anyone who uses opioids	80 (31.0)	1.06 (0.77, 1.45)	0.98 (0.71, 1.36)
Someone who uses opioids	58 (37.2)	1.27 (0.91, 1.76)	1.08 (0.75, 1.54)

^a^Adjusted for age, sex, race/ethnicity, current homelessness, and OUD severity

#### Cohabitation romantic and non-romantic partners

There was evidence that cohabitation with a romantic partner is protective against having 0% protected opioid use events (APR: 0.89, 95% CI 0.76, 1.04). Living with a partner who also uses opioids was associated with a 23% reduction in the hazard of having no protection during opioid use (APR: 0.77, 95% CI 0.62, 0.95). While there was a weak and non-significant relationship between living with anyone and protected events (APR: 0.99, 95% CI 0.85, 1.15), living with anyone who uses opioids was associated with having more than 25% more protected use events.

### Associations: network and support factors and overdose (Table 4)

#### Network size

In both unadjusted and adjusted analyses, among injectors, there was little evidence of an association between network size and overdose risk.

#### Cohabitation romantic and non-romantic partners

Cohabitation with a romantic partner, with a partner who also uses opioids, or with anyone was not associated with overdose experiences.

## Discussion

Overall, our results shed new light on social networks of people who use drugs, as participants in our study with larger social networks had greater coverage of naloxone and a person to administer it during opioid use events, what we have called “protected use,” than participants with smaller networks. Having larger social networks of people who use opioids has the potential to result in a higher proportion of protected use events. By the same token, our finding that 45% of all participants reported that naloxone was *never* available during past-month opioid use events attests to the challenges we continue to face in trying to tackle this crisis and ensure that for every use of opioids, there is a naloxone kit and someone to administer it nearby.

Participants with larger social networks were considerably more likely to have protected use events, networks that have potential to provide more support to mitigate some of the hazards associated with solitary injection drug use and overdose response—a network member can administer naloxone in a timely way. Our finding that larger networks were not associated with overdose experiences in past 30 days, but were associated with ever being trained in naloxone use and having administered the medication in the past three months, supports other research showing the positive benefits of saturating communities with naloxone and ensuring the medication makes into the hands of people who use drugs [34–36]. The comparatively low prevalence of overdose experience among people who inject drugs who have no network members may be, among other factors, an indication that people who inject drugs alone are aware of having no potentially life-saving support and use with greater caution. Especially when using alone, these people who inject drugs may more readily use smaller amounts, check drug supplies for fentanyl using test strips distributed by harm reduction agencies, and/or do “test shots” to determine potency, all features of THN curricula. Future research is needed to better understand strategies deployed by solitary users to stay safe when they use alone, and if they are more cautious in solitary use situations.

Living with anyone who uses opioids, including a romantic partner, was associated with more naloxone protection but not more overdose experiences than among those who live alone or with a non-opioid user. This is yet another indication that naloxone distribution efforts need to also focus on getting naloxone into the extended networks of people who use drugs, users and non-users. But it also suggests that more targeted outreach to non-users who may live or interact with people who use drugs is needed. That one quarter (140 participants) of our sample lived alone, 33% reported an overdose experience in the past 30 days, and 45% reported being never protected in the previous month of opioid use, suggests that the goal of being protected 100% of the time by naloxone and someone to administer it demonstrates the work ahead to connect people to protective networks and contexts for use (i.e., supervised spaces).

In the United States, the primary means of supervised drug consumption stems from one’s network and the public, as interventions like safe consumption sites (SCSs) or Overdose Prevention Centers (OPCs) remain illegal at the federal level, placing the burden of overdose response one’s social networks, often carried by people without the institutional frameworks around them, which can take an emotional toll [37]. One known underground OPC in the US has demonstrated individual and social benefits, showcasing the viability of this proven intervention in Canada and Europe within U.S. contexts [38–40]. Recently, two sanctioned OPCs began operating “above ground” in NYC communities that have experienced high levels of overdose mortality. In just the first two months of operation, the OPC reported 124 onsite overdose interventions, a strong testament to their life saving potential and the need for large scale implementation across U.S. communities so people are always able to find low threshold protection. The high rates of overdose fatalities in private residences [11] are a strong reminder that THN adaptation for solitary living, housing and shelter systems are sorely needed to assist people who use drugs, including supervised use spaces easily accessible in residences and group trainings that may have the secondary function of helping to connect people after explaining the value of having a buddy system [41]. An environment free of stigma and judgement can only benefit naloxone uptake. Based on our findings, it is clear that additional tools are needed to help people who use drugs prevent an overdose in the first place (e.g. safer drug supply programs [42]), expedite response to overdose events (e.g. SCSs [43]), and establish better mechanisms of protection for solitary users they may need in the event of an overdose (e.g. housing overdose prevention sites [44]; technology-adapted tools [45]). Decriminalization of drugs might also address fluctuations in opioid tolerance resulting from incarceration, and address the surreptitious nature of drug use shaped by criminalization, resulting in fewer solitary opioid consumption events. Additionally, innovative safety measures developed during the height of the COVID-19 pandemic may also prove useful for minimizing overdose fatalities among solitary users including telemedicine monitoring programs, technology to link friends or just fellow-users via the phone or social media, and home visitation wellness programs [46].

Our findings related to race suggests that additional research is critical to understanding why communities of people of color who use opioids appear less protected by naloxone than other groups [42]. Our finding that Black participants were considerably less likely to have protected use events may reflect an ongoing broader lack of access to the medication and understandable hesitancy to engage harm reduction resources more generally in some communities due to legacies of harm done onto communities of people of color by medical institutions [47, 48]. Black people who use opioids may face logistical and financial barriers to treatment [49] and may have had negative experiences with healthcare. These past experiences and poor quality care may reinforce mistrust, which may also be a barrier to naloxone uptake [50–52]. Limited access to naloxone may also be grounded in the history of community resistance to syringe exchange programs in some urban Black neighborhoods [46] as well as ongoing stigmatization, stereotyping, and criminal justice responses to drug use in Black communities [53]. Taken together, these phenomena point to the critical need for tailored harm reduction curricula and outreach, developed by and for communities of people of color, in addition to outreach to underserved communities guided by community members. Peer-based programs may be of particular value [48].

For those persons who are 50 years or older, while they are less likely to have to have small networks, they do not have the same protection found in the full sample, where large networks come with greater protection (or less unprotectedness). Thus, for those who are 50 or older, despite being less likely to have smaller networks, they are also less likely to be protected, which moves against the overall correlation between network size and protection. With an aging population of opioid users and a high proportion of our sample reporting solitary living, additional research is needed to better understand why larger networks don’t afford older people the same naloxone protection as found with other groups.

Our findings that participants with mental health symptoms of depression, anxiety, and suicidality were considerably more likely to have experienced an overdose in the past 30 days is consistent with recent research that shows experiencing anxiety, suicidal ideation,  and depression can be a risk factor for overdose [54, 55]. Additional overdose prevention and naloxone distribution may prove fruitful alongside mental health screenings. Also, further research on co-morbidities and overdose risk is clearly needed as are innovative outreach models to reach persons at high risk for overdose with mental health concerns.

We note several limitations in the current study, the first being the study was conducted in NYC, a city with an expansive naloxone distribution infrastructure, and the experiences of people who use opioids may not be generalizable to other areas. Data are self-report and there is the possibility of under and over reporting. Measuring non-fatal overdose is difficult and may be biased by the social contexts of use, in that those who use with others are more likely to learn of non-fatal ODs—characterized by collapse, unexpected loss of consciousness, fingers or lips turning blue, etc.—than those that occur in isolated, solitary-use settings. Our data on network size is limited to the size of participants’ opioid-using networks, we did not collect data on the full size of all of participants’ non opioid-using networks. Finally, it is possible that there is an underreporting of overdose experiences as a person may not recognize that he/she had experienced an overdose.

## Conclusion

Considering the importance of protected use amid the ongoing dual overdose and COVID-19 crises, it is vital that the high levels of unprotected use events reported in our study be disseminated and discussed among people who use drugs, particularly among people of color who use drugs. Such conversations might lead to ensuring people at risk of fatal overdose know where to get naloxone, consider who trusted peers are to use drugs with, are aware that overdose prevention sites are now available above ground in New York City, and consider what protected drug use means in their lives. Agencies providing services to people who use drugs or community-based participatory action researchers might be best positioned to host such dialogues.

In this era of fentanyl, overdoses occur more frequently and rapidly than those involving only heroin. With fentanyl detected in over half of our sample (59%) and reports of more fentanyl in the illicit drug supply, it is critical that opioid use events involve a bystander or readily available responder and easily accessible naloxone to make sure the incidence of overdose does not continue to climb. As communities face overdose fatigue, naloxone saturation remains as important as ever and our collective efforts are needed to expand the reach of naloxone distribution and help foster a climate where people carry the medication without fear of police repercussion and judgement by others [56]. Aiming to increase the naloxone community safety net is contingent on social network participation. As such, interventions to prevent and respond to overdoses particularly when using drugs alone are critical in the fight to end the fatal overdose crisis.

## Data Availability

The datasets used and/or analyzed during the current study are available from the corresponding author on reasonable request.
